# Fatores Associados aos Triggers e Eventos Adversos em Pediatria*

**DOI:** 10.15649/cuidarte.3060

**Published:** 2023-12-24

**Authors:** Rúbia Marcela Rodrigues Moraes, Sileyde Cristiane Bernardino Matos Povoas Jucá, Deise Vacario de Quadros, Ana Maria Müller de Magalhaes, Gilmar Jorge de, Welistania Vieira Bispo Barbosa, Joao Lucas Campos de Oliveira

**Affiliations:** 1 . Hospital Universitário Júlio Müller, Cuiabá, MT, Brasil. Email: rubia mc@yahoo.com.br Hospital Universitário Júlio Müller Cuiabá MT Brasil rubia mc@yahoo.com.br; 2 . Hospital Universitário Júlio Müller, Cuiabá, MT, Brasil. E-mail: mscjuca@gmail.com Hospital Universitário Júlio Müller Cuiabá MT Brasil mscjuca@gmail.com; 3 . Universidade Federal do Rio Grande do Sul, Porto Alegre, RS, Brasil. Email: deisevquadros@gmail.com Universidade Federal do Rio Grande do Sul Universidade Federal do Rio Grande do Sul Porto Alegre RS Brazil deisevquadros@gmail.com; 4 . Universidade Federal do Rio Grande do Sul, Porto Alegre, RS, Brasil. Email: amagalhaes@hcpa.edu.br Universidade Federal do Rio Grande do Sul Universidade Federal do Rio Grande do Sul Porto Alegre RS Brazil amagalhaes@hcpa.edu.br; 5 . Universidade Federal do Mato Grosso, Cuiabá, MT, Brasil. Email: gilmar.junior@ufmt.br Universidade Federal do Mato Grosso Universidade Federal do Mato Grosso Cuiabá MT Brazil gilmar.junior@ufmt.br; 6 . Universidade Federal do Mato Grosso, Cuiabá, MT, Brasil. E-mail: welistania@gmail.com Universidade Federal do Mato Grosso Universidade Federal do Mato Grosso Cuiabá MT Brazil welistania@gmail.com; 7 . Universidade Federal do Rio Grande do Sul, Porto Alegre, RS, Brasil. E-mail: joao-lucascampos@hotmail.com Universidade Federal do Rio Grande do Sul Universidade Federal do Rio Grande do Sul Porto Alegre RS Brazil joao-lucascampos@hotmail.com

**Keywords:** Eventos Adversos, Gestáo de Riscos, Rastreamento, Seguranza do Paciente, Enfermagem Pediátrica, Adverse Events, Risk Management, Tracking, Patient Safety, Pediatric Nursing, Eventos Adversos, Gestión de Riesgos, Seguimiento (Tamizaje), Seguridad del Paciente, Enfermería Pediátrica

## Abstract

**Introdujo:**

A ocorrencia frequente de eventos adversos durante a internado hospitalar demanda meios proativos de gerenciamento de riscos, incluindo a verificado de rastreadores/triggers.

**Objetivo:**

Verificar os fatores associados aos triggers e eventos adversos na internado pediátrica.

**Material e Métodos:**

Pesquisa transversal embasada na metodologia do Institute for Healthcare Improvement (IHI), por meio da aplicado do Paediatric Trigger Tool (PTT) a uma amostra (n=194) de prontuários de pacientes pediátricos de um hospital do Centro-Oeste do Brasil. Foi realizada análise estatística descritiva, inferencial e regressao de Poisson.

**Resultados:**

Mais da metade (n=107; 55,15%) dos pacientes apresentou pelo menos um trigger na internado. Foram identificados 204 triggers/gatilhos, com maior ocorrencia de queda de hemoglobina/ hematócrito (9,80%), queda de saturado de oxigenio (9,80%) e aumento de marcadores de fundes renais (9,20%). Do total de gatilhos, 64 (31,37%) eventos adversos foram confirmados, os quais foram classificados majoritariamente como dano temporário com necessidade de suporte ao paciente (65,62%). O tempo de internado (p-valor=0,004) e o caráter da internado (p-valor<0,001) foram variáveis associadas a ocorrencia de triggers. Caráter de internado e admissóes provenientes de outras instituióes foram preditores na ocorrencia de triggers e eventos adversos.

**Discussao:**

O estudo encontrou 31,37% dos triggers resultando em danos ao paciente, a detecáo precoce é essencial na seguranza do paciente pediátrico, internaóes prolongadas estáo ligadas a infecdes e eventos adversos, transferencias de pacientes exigem medidas de seguranza rigorosas e eficazes.

**Conclusóes:**

Internaóes prolongadas e crianzas admitidas via transferencia merecem atenáo a triggers e/ou eventos adversos concretizados.

## Abstract (en)

**Introduction:**

The frequent occurrence of adverse events during hospital admission demands proactive means of risk management, including checking trackers/triggers.

**Objective:**

To verify the factors associated with triggers and adverse events in pediatric hospitalization.

**Material and Methods:**

Cross-sectional research based on the Institute for Healthcare Improvement (IHI) methodology, through the application of the Pediatric Trigger Tool (PTT) to a sample (n= 194) from medical records of pediatric patients from a hospital in the Center-West of Brazil. Descriptive, inferential statistical analysis and Poisson regression were performed.

**Results:**

More than half (n=107; 55.15%) of patients had at least one trigger upon admission. 204 triggers were identified, with the highest occurrence of a drop in hemoglobin/hematocrit (9.80%), a drop in oxygen saturation (9.80%) and an increase in kidney function markers (9.20%). Of the total triggers, 64 (31.37%) adverse events were confirmed, which were mostly classified as temporary damage requiring patient support (65.62%). The length of stay (p-value=0.004) and the nature of the hospitalization (p-value<0.001) were variables associated with the occurrence of triggers. Character of hospitalization and admissions from other institutions were predictors of the occurrence of triggers and adverse events.

**Discussion:**

The study found 31.37% of triggers resulting in harm to the patient, early detection is essential in pediatric patient safety, prolonged hospitalizations are linked to infections and adverse events, patient transfers require rigorous and effective safety measures.

**Conclusions:**

Prolonged hospitalizations and children admitted via transfer deserve attention to triggers and/or adverse events.

## Resumen

**Introducción:**

La frecuente aparición de eventos adversos durante el ingreso hospitalario exige medios proactivos de gestión de riesgos, incluida la verificación de rastreadores/disparadores.

**Objetivo:**

Verificar los factores asociados a desencadenantes y eventos adversos en la hospitalización pediátrica,

**Material y Métodos:**

Investigación transversal basada en la metodología Institute for Healthcare Improvement (IHI), mediante la aplicación del Pediatric Trigger Tool (PTT) a una muestra (n= 194) de historias clínicas de pacientes pediátricos de un hospital del Centro-Oeste de Brasil. Se realizaron análisis estadísticos descriptivos, inferenciales y regresión de Poisson.

**Resultados:**

Más de la mitad (n=107; 55,15%) de los pacientes presentaron al menos un desencadenante al ingreso. Se identificaron 204 desencadenantes, con mayor incidencia de descenso de la hemoglobina/hematocrito (9,80%), descenso de la saturación de oxígeno (9,80%) y aumento de los marcadores de función renal (9,20%). Del total de desencadenantes, se confirmaron 64 (31,37%) eventos adversos, los cuales en su mayoría fueron clasificados como daños temporales que requirieron apoyo del paciente (65,62%). La duración de la estancia (p-valor=0,004) y la naturaleza de la hospitalización (p-valor<0,001) fueron variables asociadas con la aparición de desencadenantes. El carácter de la hospitalización y los ingresos de otras instituciones fueron predictores de la aparición de desencadenantes y eventos adversos.

**Discusión:**

El estudio encontró que el 31,37% de los desencadenantes resultan en daño al paciente, la detección temprana es esencial en la seguridad del paciente pediátrico, las hospitalizaciones prolongadas están vinculadas a infecciones y eventos adversos, los traslados de pacientes requieren medidas de seguridad rigurosas y efectivas.

**Conclusiones:**

Las hospitalizaciones prolongadas y los niños ingresados vía traslado merecen atención a los desencadenantes y/o eventos adversos.

## Introdujo

Na hospitalizado pediátrica, a oferta do cuidado seguro e de qualidade a crianza e/ou adolescente e família, considerando o contexto clínico, sociocultural e organizacional, permeia inúmeros desafios em virtude da maior vulnerabilidade a incidencia de eventos adversos (EA). Isso decorre de fatores intrínsecos - como estágios do crescimento e desenvolvimento - e aspectos anatómicos e fisiopatológicos peculiares a essa clientela^1^. Além disso, elementos como a organizado e gestáo do trabalho podem favorecer a ocorrencia de incidentes e EA em pediatria, evidenciados pela evitabilidade de uma elevada proporgáo destas ocorrencias^2^.

Estudo brasileiro feito no Ceará verificou uma ocorrencia de eventos adversos notificados como *never events* e óbitos em recém-nascidos com idade > 28 dias de 7,5% e crianzas com idade entre 5-11 anos de 5%^3^. Ainda no Brasil, observou-se num período de seis anos (2008-2013) 3.330 reagóes adversas em criangas, sendo que 28% dos relatos suspeitos das reagóes adversas medicamentosas envolviam menores de um ano, e quase 30% das notificagóes envolveram o uso *off label* dos medicamentos^4^. No México, em um hospital pediátrico, 81% das reagóes adversas medicamentosas foram consideradas graves, sendo 0,7% com sequelas e 1,10% evoluíram para óbito^5^.

A incidencia, gravidade e evitabilidade de EA sáo fatores que justificam o melhor gerenciamento de riscos, inclusive na hospitalizagáo pediátrica^6^. Para isso, ferramentas de investigagáo de EA e de *triggers*/gatilhos sáo instrumentos que qualificam o gerenciamento de riscos e podem colaborar para a seguranga do paciente, pediátrico ou náo.^7^*Trigger*/gatilho é definido como uma palavra sinalizadora/ rastreador ou pista que ajuda determinado revisor a encontrar um EA ou situagáo que possa favorecer a sua ocorrencia. Por sua vez, EA é um incidente que resulta em dano ao paciente^8^.

Existem variadas formas de investigagáo de EA nas instituigóes hospitalares, que sáo respaldadas por instrumentos de detecgáo, incluindo: revisáo do prontuário do paciente; análise dos índices de gravidade, mortalidade e morbidade; verificagáo dos sistemas de notificagáo voluntários; observagáo direta e a avaliagáo dos sistemas de ouvidoria^7^^-^^9^. Uma revisáo de literatura recente7 que analisou 13 estudos primários apontou que os métodos/instrumentos de revisáo retrospectiva de prontuários para avaliagáo da incidencia e evitabilidade de EA em hospitais mais frequentes foram o *Harvard Medical Practice Study, o Canadian Adverse Event Study, o Quality in Australian Health Care Study e o Global Trigger Tool.* A relevancia dessas ferramentas se dá justamente pela persistente baixa notificagáo de eventos adversos nas organizagóes de saúde, motivada especialmente pelo medo,^9^ portanto, sáo meios que podem aproximar a gestáo de riscos a realidade assistencial, que é dinamica e complexa.

Conhecer quais pacientes sáo mais suscetíveis aos *triggers* e aos EA, sensíveis ao emprego de ferramentas de rastreio, pode contribuir de forma robusta ao avango científico e clínico-gerencial no cenário do gerenciamento de riscos e seguranga do paciente pediátrico, pois se trata de uma clientela com particularidades assistenciais e maior susceptibilidade a ocorrencia de incidentes. Deste modo, este estudo teve o intuito de responder as seguintes perguntas: quais sáo os fatores associados aos *triggers*/gatilhos e aos eventos adversos na internagáo pediátrica? E, quais sáo os *triggers* associados aos eventos adversos na internagáo pediátrica? Com isso, o objetivo consistiu em verificar os fatores associados a ocorrencia de *triggers* e eventos adversos em criangas hospitalizadas.

## Materiais e Métodos

Estudo transversal, analítico e retrospectivo. Foi realizado na unidade de internado pediátrica de em um hospital universitário público de médio porte da regiáo Centro-Oeste do Brasil. A unidade estudada é referenciada no estado para tratamento de doengas crónicas, raras e infectocontagiosas e conta com 14 leitos de internado entre pacientes clínico-cirúrgicos vinculados exclusivamente ao Sistema Único de Saúde.

A populagáo foi constituida da totalidade (N=608) de prontuários físicos provenientes dos pacientes que estiveram internados no setor no ano de 2019. Para conseguir alcanzar os objetivos do estudo, calculou-se o tamanho mínimo necessário da amostra, utilizando a seguinte expressáo^10^:




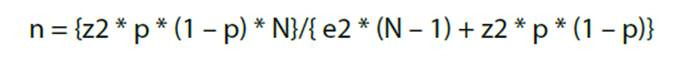




Onde, Z é obtida na tabela de distribuido normal padronizada, considerando um coeficiente de confianza de 95%, isto é, Z = 1,96; um erro de amostragem de 5,00% (e = 0,05); a proporgáo será considerado de 50% (p =0,50) para estimar o maior n possível; e o tamanho de populado N = 608. Com isso, o tamanho amostral mínimo pela equagáo aplicada foi de 236.

O processo amostral utilizado foi aleatório simples. Houve 42 perdas, com impossibilidade de reposido das unidades amostrais devido ao agravamento do contexto de pandemia de COVID-19 vigente, o que inviabilizou a continuidade da coleta de dados de in *loco*.

A coleta de dados ocorreu de outubro de 2020 a fevereiro de 2021, seguindo a metodologia do IHI.8 Para tanto, foram escolhidos de forma aleatória e proporcional 20 prontuários por mes, com pontos de cortes quinzenais. Conforme norma institucional, cada pesquisador deveria utilizar 20 prontuários físicos por vez na unidade de coleta, e o mesmo teria de solicitá-los com prazo de 72h, náo havendo com isso tempo hábil para novas coletas. Portanto, a amostra final foi constituída por 194 (82% do dimensionado) prontuários.

Os critérios de inclusáo foram: prontuários de crianzas com tempo de internado de no mínimo 24 horas e idade maior ou igual a 28 dias e menor ou igual a 16 anos, 11 meses e 29 dias; diagnóstico clínico; com desfecho: de alta hospitalar, transferencia ou óbito. Adotou-se como critérios de exclusáo: prontuários de crianzas com tempo de internado superior a seis meses (por inviabilidade de coleta de dados); prontuários incompletos em relado aos dados demográficos e clínicos; prontuários náo localizados; pacientes internados em regime de hospital-dia e pacientes cirúrgicos (objetivando uma amostra homogenea).

O rastreamento dos *triggers* e eventos adversos ocorreu por meio da aplicado do *Paediatric Trigger Tool* (PTT),^8^ e de um formulário para extrado de variáveis demográficas e clínicas dos pacientes. A ferramenta PTT contém cinco módulos constituídos por gatilhos/pistas que variam de 1 a 15 rastreadores. Os gatilhos estáo inseridos nos seguintes módulos, a saber: Cuidados gerais (11 rastreadores), Cuidados Cirúrgico (04 rastreadores), Cuidados intensivos (01 rastreador), Medicado (08 rastreadores) e Resultado de Testes Laboratoriais (15 rastreadores)

Considerado como uma variado do Global Trigger Tool (GTT) mais apropriada a clientela pediátrica, o *Paediatric Trigger Tool* (PTT) é uma ferramenta estruturada de revisáo de prontuários recomendada pelo *Institute for Healthcare Improvement* (IHI) que busca mensurar a ocorréncia de danos nas instituigóes hospitalares usando gatilhos específicos para pacientes pediátricos para identificar os *triggers* de EA, bem como confirmar a ocorréncia desses eventos.^8^ O PTT fornece as equipes pediátricas métricas quanto a incidéncia de danos relacionados a assisténcia, permitindo gerenciar fragilidades e implementar melhorias na seguranza e qualidade assistencial, acompanhando sistematicamente as estratégias propostas^8^.

Pelo perfil bem delimitado da clientela pediátrica em estudo, que tem especificidade clínica nao crítica, foram utilizados os rastreadores dos módulos: Cuidados Gerais, Medicagáo e Resultados de Testes Laboratoriais. Os módulos de Cuidados intensivos e Cuidado Cirúrgico nao foram utilizados também pelo perfil dos pacientes da unidade (poucos pacientes cirúrgicos) e do hospital, que náo possui Unidade de Terapia Intensiva Pediátrica.

Foram consideradas como variáveis dependentes os rastreadores (*triggers*) identificados pelo PTT, os eventos adversos confirmados e gravidade do dano ao paciente. Foram delimitadas como variáveis independentes: idade, sexo, diagnóstico médico principal (agrupado por categorias de acordo com CID-10 - *Classificagao Internacional de Doengas)* tempo de permanencia, caráter da internado (eletiva, emergencial ou transferencia) e desfecho clínico (alta médica, transferencia ou óbito). Para obtengáo destas variáveis considerou-se a revisáo retrospectiva de prontuários físicos de forma aleatória conforme preconizado pelo IHI.

Em conformidade a metodologia do IHI,^8^ a equipe de coleta de dados foi constituída por duas enfermeiras mestrandas e uma médica pediatra, as quais foram previamente treinadas, através da plataforma de curso *online* fornecido pelo IHI, com aspectos teóricos dos métodos e quanto as instrugóes de aplicagáo. Devido a complexidade de análise de eventos adversos, e, para confrontar dados colhidos pelos revisores bem como a dificuldade em diferenciar os EA com eventos do curso natural da doenga e/ou das morbidades apresentadas pelo paciente, necessita-se de um profissional médico, que atua na revisáo dos formulários de rastreadores, conforme preconizado pela ferramenta^11^. Portanto, a médica pediatra da equipe de coleta, em acordo com o PTT, realizou as validagóes das avaliagóes.

Antes da coleta de dados foi realizado teste piloto, em agosto de 2020, com toda a equipe de pesquisa com prontuários que náo foram utilizados no banco de dados. Neste teste, foram identificadas inconsistencias quanto a forma de preenchimento da planilha, que foram ajustadas e dúvidas foram sanadas de forma consensual e coletivamente. O instrumento de coleta de dados utilizado foi uma planilha *Excel®* elaborada pela pesquisadora com os dados pertinentes da ferramenta do PTT e as variáveis supracitadas. De acordo com a metodologia empregada, quando rastreado e confirmado um EA, há uma classificagáo de tipos de danos relacionados a assistencia, através da utilizagáo do *National Coordinating Council (NCC) for Medication Error Reporting and Prevention* (MERP),^12^ que classifica um erro de acordo com a gravidade do resultado, divididos em cinco categorias: *Categoria E:* dano temporário ao paciente e intervengáo necessária; *Categoria F:* dano temporário ao paciente e requerimento inicial ou prolongado de hospitalizagáo; *Categoria G:* dano permanente ao paciente; *Categoria H:* intervengáo necessária para sustentar a vida; *Categoria I:* morte do paciente^12^.

Para verificar as associagóes entre os desfechos e as variáveis independentes, foi considerada a razáo de prevalencia bruta com seus respectivos intervalos de confianga e o teste de qui-quadrado. Posteriormente, determinou-se as razóes de prevalencias ajustadas, utilizando o modelo de regressáo de Poisson múltiplo com variancia robusta, onde as variáveis que apresentaram no teste de qui- quadrado valor p < 0,20 foram inseridas inicialmente no modelo, e somente as que tiveram valor p < 0,05 permaneceram nele após a análise. Em virtude de algumas variáveis nao terem apresentado distribuiao em normalidade, optou-se por utilizar por testes nao paramétricos e o modelo de regressao de Poisson com variancia robusta. Todas as análises estatísticas foram realizadas com auxilio dos *softwares Rstudio®* versao 2022.7.2.0 e *Stata®* versao 14, sendo todos os intervalos com confianza de 95% e os testes com nível de significancia a 5%, sendo os dados armazenados em Zenodo^13^.

Foram cumpridas todas as normas de ética em pesquisas vigentes no Brasil, dispostas pela Resolugáo do Conselho Nacional de Saúde n° 466/2012. O projeto foi aprovado pelo Comité de Ética em Pesquisa (CAEE: 07626019.5.0000.5541) da instituido na qual o estudo foi realizado e aprovado sob parecer 3.603.794/2019.

## Resultados

As características dos pacientes (n=194) quanto ao sexo, idade e motivo de internado sao descritas na Tabela 1. Na análise de associagao das variáveis sócioclínicas em relado a ocorréncia ou nao de *triggers,* se apresentaram significativas as variáveis: tempo de internado, caráter de internado e desfecho.

A Tabela 1 apresenta as características dos pacientes quanto ao sexo, idade e quadro clínico. Na análise de associado das variáveis sócio clínicas em relado a ocorréncia ou nao de *triggers,* se apresentaram significativas as variáveis tempo de internado, caráter de internado, tipo de alta e severidade.

**Tabela 1:** Características sócioclínicas de pacientes pediátricos hospitalizados, quanto a ocorréncia ou nao de triggers. Centro-Oeste, Brasil, 2019 (n=194)

Variáveis	Sim			Nao	
	%	(n=107)	%	(n=87)	valor-p
Sexo					0,4076
Feminino	49,53	(53)	42,53	(37)	
Masculino	50,47	(54)	57,47	(50)	
Idade					0,5636
Maior e igual a 6 anos	47,66	(51)	42,99	(51)	
Menor que 6 anos	52,34	(56)	38,32	(56)	
Tempo de Internado					0,004
Maior e igual a 6 dias	60,75	(65)	39,08	(34)	
Menor que 6 dias	39,25	(42)	60,92	(53)	
CID Internado					0,9203
Urinário	24,30	(26)	24,14	(21)	
Respiratório	7,48	(8)	3,45	(3)	
Autoimune e/ou genético	12,15	(13)	9,20	(8)	
Gastrointestinal	11,21	(12)	11,49	(10)	
Neurológico	5,61	(6)	11,49	(10)	
Cardiovascular	22,43	(24)	24,14	(1)	
Endócrino	7,48	(8)	9,20	(8)	
Metabólico	4,67	(5)	3,45	(3)	
Violencia Sexual	1,87	(2)	1,15	(1)	
Tegumentar	0,93	(1)	1,15	(1)	
Infeccioso	1,87	(2)	1,15	(21)	
Caráter da internado					< 0,001
Eletivo	4,67	(5)	20,69	(18)	
Transferido	23,36	(25)	10,34	(9)	
Urgencia/Emergencia	71,96	(77)	68,97	(60)	
Desfecho					0,010
Alta Melhorada	85,05	(91)	97,70	(85)	
Transferencia	14,02	(15)	2,30	(2)	
Óbito	0,93	(1)	0,00	-	

*Fonte: dados da dissertagáo de mestrado da autora; teste estatístico de qui-quadrado para cálculo dep-valor.*

Do total de pacientes (n=194), 55,15% e 22,16% apresentou, respectivamente, pelo menos um *trigger e/ou* evento adverso na sua hospitalizado. A Tabela 2 ilustra as medidas quantitativas dos pacientes que apresentaram ou nao *triggers* e eventos adversos.

**Tabela 2:** Mínimo, máximo, mediana, intervalo interquartil para as variáveis quantitativas: idade, tempo de internado e a ocorrencia ou nao de Triggers, eventos adversos e grau de severidade de eventos adversos entre pacientes pediátricos hospitalizados. Centro-Oeste, Brasil, 2019 (n=194)

Variáveis	Pacientes com Triggers							
	Sim (n=107)				Nao (n=87)			
	Min	Max	Mediana	Q3 - Q1 (IQR)	Min	Max	Mediana	Q3 - Q1 (IQR)
Idade em anos	1	16	5,00	10,00 - 2,00 (8)	1	16	5,00	11,00 - 2,00 (9)
Tempo Total de Internado	1	74	7,00	11,50 - 4,00 (7,5)	1	126	5,00	8,50 - 3,00 (5,5)
Variáveis				Pacientes com Eventos Adversos				
			Sim (n=43)				Nao (n=	151)
	Min	Max	Mediana	Q3 - Q1 (IQR)	Min	Max	Mediana	Q3 - Q1 (IQR)
Idade em anos	1	16	4	10,00 - 2,50 (8,5)	1	16	6,00	11,00 - 2,00 (9)
Tempo Total de Internado	1	74	10,00	14,50 - 4,50 (10)	1	126	5,00	9,00 - 3,00 (6)
Grau de Severidade	0	6	2,00	3,50 - 2,00 (1,5)	0	3	0,00	1,00 - 0,00 (1)

* IQR - Intervalo interquartil: Q3,3° quartil (superior, 75%) e Q1,1°quartil (inferior, 25%).

No total da amostra de pacientes (n=194) foram identificados 204 *triggers*/gatilhos de eventos adversos, ou seja, vários pacientes apresentaram mais de um *trigger.* Destes, 64 (31,37%) eventos adversos foram confirmados.

A Tabela 3 apresenta a frequéncia dos tipos de *triggers* por módulos do PTT. Verifica-se que os *triggers* com maior ocorréncia foram queda de hemoglobina, queda de saturado e aumente.

**Tabela 3:** Frequência de triggers na hospitalização pediátrica, por módulos do Paediatric Trigger Tool (PTT), Centro-Oeste, Brasil, 2019.o de marcadores renais

*Triggers* Identificados por módulos do PTT	% (n)	% (n)
Cuidados Gerais	100 (66/66)	32,35 (66/204)
Danos teciduais	18,18 (12)	5,88 (12)
Readmissao hospitalar < 30 dias	3,03 (2)	0,98 (2)
Admissao nao planejada	1,52 (1)	0,49 (1)
Imagem craneana anormal	3,03 (2)	0,98 (2)
Parada cardíaca e/ou respiratória	1,52 (1)	0,49 (1)
Diag. embolia / trombose pulmonar	1,52 (1)	0,49 (1)
Complicates	19,70 (13)	6,37 (13)
Transferencia	21,21 (14)	6,86 (14)
SPO2 < 85%	30,30 (20)	9,80 (20)
Medicamentos	100 (38)	18,63 (38/204)
Vit K exceto de rotina em RN	28,95 (11)	5,39 (11)
Glucagon / Glicose 10%	2,63 (1)	0,49 (1)
Anti-histamínicos	10,53 (4)	1,96 (4)
Antieméticos	7,89 (3)	1,47 (3)
Colóide ou cristalóide em bolus	23,68 (9)	4,41 (9)
Suspensao abrupta da medicado	26,32 (10)	4,90 (10)
Resultado de Testes Laboratoriais	100 (100)	49,02(100/204)
INR >5 ou APTT>99	2,00 (2)	0,98 (2)
Transfusao	14,00 (14)	6,86 (14)
Queda >25% de Hb ou Hct	20,00 (20)	9,80 (20)
Uréia e Creatinina >2x basal	19,00 (19)	9,31 (19)
Na+ <130 or >150	4,00 (4)	1,96 (4)
Hipoglicemia (<3mmol/L ou <54mg/dL)	9,00 (9)	4,41 (9)
Hiperglicemia (>12mmol/L ou 216 mg/dL)	13,00 (13)	6,37 (13)
Pneumonia nosocomial	3,00 (3)	1,47 (3)
Plaquetas < 100.000	6,00 (6)	2,94 (6)
Cultura de sangue positiva	2,00 (2)	0,98 (2)
Evento nao identificado por trigger	8,00 (8)	3,92 (8)

Em relagáo a severidade dos eventos adversos confirmados (n=64), os mais expressivos foram os da categoría E (65,62%), ou seja, levaram a dano temporário ao paciente com intervengo necessária (Tabela 4).

**Tabela 4:** Grau de severidade dos Eventos Adversos (EA) confirmados entre pacientes pediátricos hospitalizados. Centro-oeste, Brasil, 2019. (n=64)ais

Grau de Severidade do EA	%	n(64)
E = Dano temporario ao paciente e intervengo necessária	65,62	42
F = Dano temporário ao paciente e requerimento inicial ou pro-	31,25	20
longado de hospitalizacao		
G = Dano permanente ao paciente	-	-
H = Intervengo necessária para sustentar a vida	3,12	2
I = Óbito	-	-

Foram realizadas as análises com as variáveis sexo, idade, que foram utilizadas como ajuste, tempo de internado e caráter de internado. Assim, a Tabela 5 demonstra tempo de internado e caráter de internagáo para ocorréncia de *triggers* e também como preditores de evento adverso.

**Tabela 5:** Razao da Prevalencia Bruta (R.P.B) e Razao da Prevalencia Ajustada (R.P.A), para ocorréncia de triggers e eventos adversos e as variáveis socioclínicas de pacientes pediátricos hospitalizados, Centro Oeste, Brasil, 2019

Variáveis	Sim	Nao	valor-p*	R.P.B		R.P.A	
	% (n=107)	% (n=87)		R.P [IC(95%)]	valor-p**	R.P [IC(95%)]	valor-p**
Sexo			0,4076				
Masculino	50,47 (54)	57,47 (50)		1			
Feminino	49,53 (53)	49,53 (37)		0,85 [0,62; 1,17]	0,336		
Idade			0,5636				
Menor que 6 anos	52,34 (56)	38,32 (41)		1			
Maior e igual a 6 anos	47,66 (51)	42,99 (46)		1,12 [0,82; 1,53]	0,472		
Tempo de Internado			0,004				
Maior ou igual 6 dias	60,75 (65)	39,08 (34)		1		1,00	
Menor ou igual a 6 dias	39,25 (42)	60,92 (53)		1,62 [1,17; 2,25]	0,004	1,44[1,04; 2,02]	0,030
Caráter da internado			< 0,001				
Eletivo	4,67 (5)	20,69 (18)		1		1,00	
Transferido	23,36 (25)	10,34 (9)		0,54 [0,30; 0,97]	0,040	0,39 [0,21;0,72]	0,003
Urgéncia/Emergéncia	71,96 (77)	68,97 (60)		0,92 [0,66; 1,29]	0,645	0,61 [0,46; 0,80]	< 0,001
Evento Adverso	% (n=43)	% (n=151)					
Sexo			0,3961				
Masculino	60,46 (26)	51,65 (78)		1			
Feminino	39,53 (17)	48,34 (73)		1,81[0,93;1,25]	0,305		
Idade			0,2967				
Menor que 6 anos	41,86 (18)	52,32 (79)		1			
Maior e igual a 6 anos	58,14 (25)	47,68 (72)		0,91[0,78;1,06]	0,229		
Tempo de Internado			0,1151				
Maior que 6 dias	62,79 (27)	47,68 (72)		1			
Menor que 6 dias	37,21 (16)	52,32 (79)		1,14[0,98;1,33]	0,082		
Caráter da internado			0,0525				
Eletivo	4,65 (2)	13,91 (21)		1		1	
Transferido	27,91 (12)	14,57 (22)		1,20[1,031;1,39]	0,018	0,70[0,53;0,94]	0,016
Urgéncia/Emergéncia	67,44 (29)	71,52 (108)		0,80[0,62;1,04]	0,098	0,86[0,74;1,006]	0,061

* valor-p do teste estatístico qui-quadrado;prevalencia bruta e razáo de prevalencia ajustada. ** valor-p para razáo de.

Na análise de regressáo de Poisson múltipla, as variáveis que apresentaram significancia com valor-p < 0,05 foram: tempo de internado e caráter de internado, sendo que o tempo de internado menor que 6 dias foi de 1,44 vezes a prevaléncia do tempo de internado menor que 6 dias para ocorréncia de *trigger.* O caráter de internado por transferencia foi de 0,39 vezes a prevaléncia em relagáo ao caráter de internado de forma eletiva, e o caráter urgencia e emergencia foi de 0,61 vezes a prevaléncia do caráter de internado de forma eletiva para ocorréncia de *trigger.*

Já para a ocorréncia de evento adverso, o caráter de internado por transferéncia foi de 0,70 vezes a prevaléncia do caráter de internado de forma eletiva.

## Discussao

Verificou-se que, do total de 204 *triggers* apurados, 64 (31,37%), trataram-se de incidentes que causaram algum dano ao paciente. Esse achado corrobora com estudo retrospectivo indiano de avaliagáo de gatilhos em prontuários de pacientes pediátricos, utilizando a metodologia adaptada do GTT, que detectou 35% de eventos adversos confirmados.14 Atrelando o achado do presente estudo a literatura correlata, concorda-se com o pressuposto de que a detecgáo precoce de incidentes de seguranza do paciente é um dos pilares do cuidado seguro em saúde, até mesmo porque eventos adversos envolvem processos gerenciais, terapéuticos e medicamentosos^2^.

Acerca dos danos dos EA, a maior parte foi classificada na categoria de menor gravidade. Resultado este semelhante ao estudo realizado nos Estados Unidos, onde foi aplicado a metodologia de *Global Assessment of Pediatric Patient Safety* (GAPPS), o qual evidenciou 52,7% dos incidentes determinados nesta mesma categoria^6^. Outro estudo indiano utilizando o GTT adaptado, detectou níveis de severidades distribuídos nas categorias E (58,80%), F (23,62%) G (12,08%), H (4,95%) e I (0,55%)^10^. Considerando a literatura de base e que essa classificagáo leva em conta um nivel crescente de gravidade do evento adverso, percebe-se que na amostra estudada foi pouco frequente eventos de maior severidade, todavia, 3,12% dos eventos demandaram intervengo necessária para sustentar a vida, o que já é suficiente para justificar as políticas, programas de rastreamento e estratégias de seguranza do paciente pediátrico.

Reforja a alusáo anterior uma pesquisa realizada em um hospital pediátrico em Otawa, que demonstrou que 87,9% do total de 29 crianzas internadas sofreu um EA considerado evitável com prolongamento de sintomas relacionados, tendo como causas principais os problemas gerenciais e erros terapéuticos^2^. Neste sentido, um estudo de revisáo integrativa na área da qualidade assistencial e seguranza do paciente indica o amplo uso de ferramentas que melhorem a cultura de seguranza e o gerenciamento de riscos nas instituyes, visto que ainda existem fragilidades no cuidado seguro nas unidades de saúde^15^.

Estudos de revisáo de prontuários tém se mostrado eficazes na detecgáo de eventos adversos e atualmente sáo considerados o “padráo ouro” para identificar incidentes com danos, sendo a metodologia *Paediatric Trigger Tool* na investigado de eventos adversos através do uso dos rastreadores, na busca de incidentes com danos^7^. Na análise dos prontuários deste inquérito, queda de hemoglobina, queda de saturado e aumento de marcadores das fungóes renais foram os *triggers* prevalentes, aspecto este vai ao encontro aos resultados de um estudo realizado em um hospital infantil argentino utilizando a ferramenta GTT, que evidenciou triggers relacionados ao cuidado: (58,3%); ao uso de medicamentos: (26,78%); e a microbiologia: (14,88%), sendo os EAs mais comuns a queda de saturagáo, complicates do procedimento, uso de antieméticos e hemocultura positivo^16^. Uma recente revisáo de literatura relatou que a queda de hemoglobina pode ocorrer até o 3° dia de internado e que este é um evento multifatorial, tendo como possíveis causas: a constante retirada de sangue para exames, diluigáo do sangue dos pacientes a administrado medicamentos e uma produjo de eritrócitos reduzida, caracterizando-se como um evento adverso^17^.

Os biomarcadores renais elevados sinalizam a possibilidade de lesáo renal e prejuízo na fundo dos rins. Existe uma infinidade de fármacos considerados nefrotóxicos que devem usados com cautela aos portadores de doenga renal crónica. Neste aspecto, o aumento dos níveis séricos de creatinina e ureia, pode ser caracterizado como um evento adverso medicamentoso caso terapia utilizada seja inadequada^18^.

Dentre a maior frequéncia de *trigger* durante a internado, verificou-se pacientes com diagnóstico relacionado ao sistema urinário, os quais, muitas vezes sáo acometidos por circunstancias crónicas complexas prévias e a fragilidade orgánica renal, que os torna mais susceptíveis a danos^18^.

Para além da complexidade imposta pelo próprio processo de crescimento e desenvolvimento das crianzas, um estudo realizado em hospital mexicano em locais de alta complexidade evidenciou uma maior ocorréncia de incidentes relacionados a fatores como: excesso de trabalho, inclusive o burocrático, falta de adesáo a protocolos, falta de experiéncia e habilidades dos profissionais, que fomentam um ambiente propicio a sua ocorréncia^19^.

Dentre os grupos diagnósticos elencados, as doengas cardiovasculares (22,43%) tiveram destaque neste estudo, pois tratam-se de doengas de cunho deletério que ainda ocupam lugar de destaque nas morbidades dentre os problemas de saúde pública, no Brasil, as Doengas Crónicas náo Transmissíveis (DCNT) sáo responsáveis por uma alta proporgáo de óbitos, notadamente entre os individuos de menor renda e escolaridade. A prevaléncia dessas doengas está ligada ao consumo crescente de alimentos ultra processados, sedentarismo e obesidade infantil. Essas condigóes, por sua vez, aumentam o risco de dislipidemia, hipertensáo, diabetes e doengas cardiovasculares. O enfrentamento eficaz das DCNT demanda abordagens multidisciplinares e preventivas^20^.

Evidenciou-se neste estudo período de internagáo superior a seis dias, o que é preocupante, sendo que o maior o tempo de hospitalizagáo foi associado a maior a chance de incidentes como infecgóes de corrente sanguínea e do trato urinário, podendo resultar em danos para o paciente^6^. Essa alusáo reforga que planejamento da alta e a articulagáo do hospital com a rede de saúde é relevante para galgar níveis mais elevados de seguranga ao cuidado do paciente pediátrico^1^.

Ainda sobre o tempo extra de internagáo (> de 20 dias), um estudo nigeriano retrospectivo realizado em trés hospitais de ensino, reforgou a necessidade de maior rotatividade de leitos para otimizagáo da produgáo de servigos de saúde, visto que, quanto maior o tempo de permanéncia hospitalar maior é o risco para infecgóes, eventos adversos e morbimortalidade^21^.

No Brasil, um estudo retrospectivo realizado em dois hospitais gerais públicos e de ensino revelou que 99% dos EA poderiam ter sido evitados. Esses EA resultaram em um aumento de 65,7% no tempo de internagáo dos pacientes e foram responsáveis por 11,8% das reinternagóes^22^. Essa alusáo reforga a importáncia de investimento em estratégias proativas de gestáo de riscos, ao exemplo do rastreamento dos gatilhos, já que, conforme os dados do presente estudo, uma parcela considerável de *triggers* foi confirmada como EA.

É possível que pacientes provenientes de outras instituigóes, tenham maiores chances da ocorréncia de *triggers* quando comparados aos pacientes eletivos, visto a carga de incidentes já ocorridos em outros servigos diante de outras condutas assistenciais e o próprio itinerário prolongado nos servigos de saúde e visto as falhas nas atividades administrativas. A literatura ressalta a existéncia de um maior risco de eventos adversos durante a transferéncia de pacientes,^23^ sendo mais agravante naqueles que possuem algum tipo de instabilidade.

Em virtude do potencial danoso das admissóes de pacientes provenientes de outras instituigóes, reforga-se a necessidade da implementagao de meios/instrumentos que possam garantir a seguranza no processo de transigao do cuidado do paciente pediátrico,^24^ mediados por agóes efetivas associadas a adogao de barreiras de seguranga ao sistema da assisténcia que possam prevenir situagóes de risco e eventos adversos^23^. Razao pela qual tem sido, cada vez mais, utilizado documentos estruturados, quando da transferéncia de pacientes, visando incidir na cultura de seguranga organizacional^25^. No contexto pediátrico isso é muito relevante porque pode aumentar os riscos de eventos como: descompensagóes hemodinámicas ou ventilatórias, pelo manejo que envolve o transporte, dependendo do grau de disfungao orgánica apresentada pelo paciente.

A gravidade clínica das internagóes em caráter de urgéncia e/ou emergéncia requer da equipe cuidados especializados, ressaltando-se o perfil de acometimento crónico dos pacientes do hospital de inquérito como agravante para maior ocorréncia de EAs. Um estudo canadense realizado em 6 hospitais pediátricos de grande porte evidenciou maior ocorréncia de EA em unidades de urgéncia e emergéncia, ressaltando a importáncia em se conhecer e elaborar estratégias que visem a diminuigao da incidéncia de EAs através de práticas centradas na seguranga do paciente e na minimizagao de danos, com fomento a cultura de seguranga do paciente^2^.

A maior probabilidade de ocorréncia de eventos adversos em pacientes provenientes de outras instituigóes possivelmente decorre da complexidade do processo de transferéncia, sendo sujeito a inúmeras falhas, por estar relacionado a continuidade do cuidado. A implementagao de agóes e protocolos eficazes associados a barreiras de seguranga podem prevenir situagóes de risco e possíveis incidentes com danos, decorrendo na redugao de incidentes e óbitos relacionados a eventos adversos efeitos nas transferéncias de cuidado^26^.

A internagao menor ou igual a 6 dias, foi evidenciada como fator preditor (1,44 vezes) para ocorréncia de *Triggers* em detrimento da internagao maior ou igual a 6 dias, a redugao do tempo de permanéncia, pode diminuir a ocorréncia de eventos adversos, e está indicada desde que nao haja riscos para o paciente com o intuito de evitar alta precoce e readmissao^27^, já que a internagao prolongada agrega inúmeros riscos como infecgóes nosocomiais, resisténcia bacteriana, e mortalidade^28^.

O uso de ferramentas de rastreio de gatilhos auxilia profissionais de saúde e gestores a identificarem fragilidades e tragarem estratégias de melhoria da qualidade nos processos assistenciais,^7^ qualificado o processo assistencial e gerencial. A ferramenta PTT é um importante instrumento de gestao da qualidade assistencial, descrito na literatura internacional como uma ferramenta confiável para identificar *triggers* e eventos adversos no contexto pediátrico^8^.

### Contributes para o Campo da Enfermagem

A utilizagao da ferramenta PTT é de grande valia para enfermagem e saúde, pois racionaliza a gestao de riscos e, com isso, pode favorecer o cuidado pediátrico mais seguro. Conhecendo de forma sistemática os riscos de *triggers e* eventos adversos inerentes a clientela, o enfermeiro pode implementar a gerencia do cuidado de forma mais assertiva.

### Limitares do Estudo

Apesar de nao ser uma escala psicométrica e sim uma ferramenta com elementos clínicos muito consolidados, ou seja, que nao cabem necessariamente julgamento/opiniáo, nao foi encontrado uma versao traduzida e adaptada do *Paediatric Trigger Tool* para a língua portuguesa e em uso no Brasil, e isso, ainda que seja uma limitagáo do estudo, é também uma sinalizagáo para futuras investigares.

A possível subestimado dos *triggers* verificados, em virtude da má qualidade de alguns registros consiste em uma limitado deste estudo, um possível viés que possa existir, trata-se de um viés sistemático existam vieses sistemáticos com base no diagnóstico médico por exemplo, idade ou outra característica na detecgáo de um evento adverso por um revisor humano durante a coleta de dados usando a ferramenta. A análise de regressao multivariável também seria pertinente para confirmar a predido do fato caráter da internado. No entanto, a densidade de dados, inovado do estudo e potencial de instrumentando a respeito da gestáo de riscos em pediatria, possivelmente, superam essas limitagóes.

Todavia, apesar de ser outra limitado deste estudo, náo ter investigado outros aspectos relacionados ao cuidado e ao trabalho na unidade pediátrica de inquérito, considera-se que as informagóes advindas deste tipo de análise podem ser incrementadas por outras como, por exemplo, os recursos disponíveis, a carga de trabalho dos profissionais, a complexidade dos pacientes e o ambiente de prática profissional.

## Conclusao

Conclui-se que o tempo de internagáo < 6 dias e o caráter da internagáo via urgencia/emergencia foram associados a ocorrencia de *triggers* na hospitalizagáo pediátrica. A transferencia foi ainda um preditor a confirmagáo ocorrencia de eventos adversos. Portanto, internagóes prolongadas e criangas admitidas via transferencia, além daquelas que ingressam no hospital de forma náo eletiva, ou seja, via urgencia/emergencia, merecem atengáo a ocorrencia de *triggers* e/ou eventos adversos concretizados.

Os *triggers* prevalentes foram queda de hemoglobina ou hematócrito, queda de saturagáo de oxigenio e aumento de marcadores de fungóes renais. Em torno de um tergo do total de *triggers* apurados foram confirmados como eventos adversos, o que é uma evidencia importante na gestáo proativa de riscos assistenciais em pediatria. Quanto o grau de severidade dos eventos adversos confirmados, os de dano temporário na crianga/adolescente, com necessidade de intervengáo, foram os de maior concentragáo.
